# Prospective deep learning–based quantitative assessment of coronary plaque by computed tomography angiography compared with intravascular ultrasound: the REVEALPLAQUE study

**DOI:** 10.1093/ehjci/jeae115

**Published:** 2024-05-03

**Authors:** Jagat Narula, Thomas D Stuckey, Gaku Nakazawa, Amir Ahmadi, Mitsuaki Matsumura, Kersten Petersen, Saba Mirza, Nicholas Ng, Sarah Mullen, Michiel Schaap, Jonathan Leipsic, Campbell Rogers, Charles A Taylor, Harout Yacoub, Himanshu Gupta, Hitoshi Matsuo, Sarah Rinehart, Akiko Maehara

**Affiliations:** Heart & Vascular Institute, McGovern Medical School, 1825 Pressler Street, SRB 205A, Houston, TX 77030, USA; Heart & Vascular, LeBauer-Brodie Center/Cone Health Heart and Vascular, Greensboro, NC, USA; Department of Medicine, Kindai University, Osaka, Japan; Cardiology, Icahn School of Medicine at Mount Sinai, New York, NY, USA; Cardiology, Cardiovascular Research Foundation, New York, NY, USA; HeartFlow, Inc., Mountain View, CA, USA; HeartFlow, Inc., Mountain View, CA, USA; HeartFlow, Inc., Mountain View, CA, USA; HeartFlow, Inc., Mountain View, CA, USA; HeartFlow, Inc., Mountain View, CA, USA; Radiology, University of British Columbia, Vancouver, Canada; HeartFlow, Inc., Mountain View, CA, USA; HeartFlow, Inc., Mountain View, CA, USA; Cardiology, Northwell Health Staten Island University Hospital, New York, NY, USA; Radiology, Valley Health System, Ridgewood, NJ, USA; Medicine, Gifu Heart Center, Gifu, Japan; Cardiology, Charleston Area Medical Center Memorial Hospital, Charleston, WV, USA; Cardiovascular Research Foundation, Columbia University, New York, NY, USA

**Keywords:** coronary artery disease, coronary luminal stenosis, vulnerable plaque, acute coronary syndrome, artificial intelligence, machine learning

## Abstract

**Aims:**

Coronary computed tomography angiography provides non-invasive assessment of coronary stenosis severity and flow impairment. Automated artificial intelligence (AI) analysis may assist in precise quantification and characterization of coronary atherosclerosis, enabling patient-specific risk determination and management strategies. This multicentre international study compared an automated deep learning-based method for segmenting coronary atherosclerosis in coronary computed tomography angiography (CCTA) against the reference standard of intravascular ultrasound (IVUS).

**Methods and results:**

The study included clinically stable patients with known coronary artery disease from 15 centres in the USA and Japan. An AI-enabled plaque analysis was utilized to quantify and characterize total plaque (TPV), vessel, lumen, calcified plaque (CP), non-calcified plaque (NCP), and low-attenuation plaque (LAP) volumes derived from CCTA and compared with IVUS measurements in a blinded, core laboratory-adjudicated fashion. In 237 patients, 432 lesions were assessed; mean lesion length was 24.5 mm, and mean IVUS-TPV was 186.0 mm^3^. AI-enabled plaque analysis on CCTA showed strong correlation and high accuracy when compared with IVUS; correlation coefficient, slope, and Y intercept for TPV were 0.91, 0.99, and 1.87, respectively; for CP volume 0.91, 1.05, and 5.32, respectively; and for NCP volume 0.87, 0.98, and 15.24, respectively. Bland–Altman analysis demonstrated strong agreement with little bias for these measurements.

**Conclusion:**

AI-enabled CCTA quantification and characterization of atherosclerosis demonstrated strong agreement with IVUS reference standard measurements. This tool may prove effective for accurate evaluation of coronary atherosclerotic burden and cardiovascular risk assessment.

## Graphical Abstract

**Graphical Abstract:**
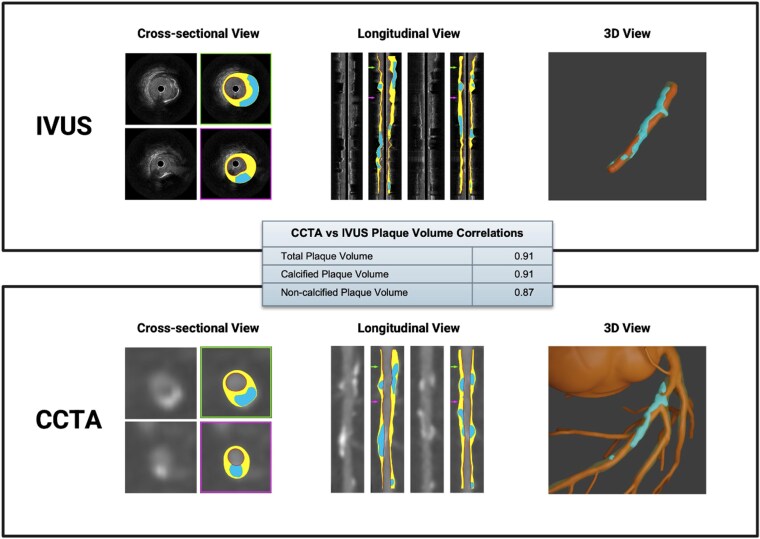
Matching cross-sectional, longitudinal, and 3D views of IVUS and CCTA show correlating presence of calcified and non-calcified plaque between the two imaging modalities. The table highlights plaque volume correlations for total plaque (TPV), calcified plaque (CP), and non-calcified plaque (NCP) on a per-lesion basis (*n* = 432). In the cross-sectional and longitudinal view, blue-coloured areas are calcified plaque and yellow-coloured areas are non-calcified plaque. In the 3D view, blue-coloured area is calcified plaque and yellow translucent-coloured area is non-calcified plaque.

## Introduction

Coronary computed tomography angiography (CCTA) has recently received a Class 1 recommendation with Level A evidence by the American College of Cardiology (ACC) and American Heart Association (AHA) for the evaluation of symptomatic patients with suspected coronary artery disease (CAD).^1^ Historically, the CCTA was proposed as the non-invasive diagnostic test for the exclusion of obstructive coronary disease and visual identification or exclusion of atherosclerosis. Subsequently, CCTA was demonstrated to define the functional significance of luminal stenosis by non-invasively calculating fractional flow reserve (FFR_CT_) at rest^2,3^ and reported to allow for the visual identification of high-risk plaques^4^ characterized by presence of low-attenuation plaque (LAP) and positive remodelling (PR).^5,6^ Clinically, plaque characterization may inform patient-specific risk stratification and management strategies. While visual assessment of coronary plaque appearance is considered an important component of a contemporary CCTA report, quantification of total atherosclerosis burden has been limited as manual plaque segmentation has proven challenging for time requirements and limited inter- and intra-observer reproducibility.^7^

New artificial intelligence (AI)-enabled automated CCTA plaque segmentation tools have been developed which may provide more rapid and reproducible quantification of atherosclerosis, but there are limited data evaluating the agreement of these tools with an accepted reference standard such as IVUS. To that end, the international pRospEctiVe, multicEnter study to AnaLyze PLAQUE (REVEALPLAQUE) study (ClinicalTrials.gov identifier: NCT05138289) sought to evaluate the level of agreement between AI-enabled quantitative coronary plaque analysis (AI-QCPA) and IVUS.

## Methods

### Study population

Fifteen centres in the USA and Japan participated in the REVEALPLAQUE study, approved by the Western and Copernicus Group Institutional Review Board (WCG-IRB) in September 2021. Sites enrolled clinically stable patients, 18 or older, with known CAD, a CCTA showing luminal stenosis in at least one major epicardial coronary vessel of stentable/graftable diameter, and successfully processed FFR_CT_ analysis (HeartFlow, Mountain View, CA) in whom clinically indicated invasive coronary angiography (ICA) and IVUS were planned within 45 days after CCTA. CT acquisitions were performed across a wide range of CT scan platforms and across varied tube potentials with images reconstructed using a wide range of iterative and filtered back projection algorithms (Supplementary appendix). Patients with acute chest pain, a history of percutaneous coronary intervention (PCI) or coronary artery bypass graft (CABG) surgery prior to CCTA acquisition, myocardial infarction (MI) within 30 days before CCTA, suspicion of acute coronary syndrome between CCTA and ICA, known complex congenital heart disease, and tachycardia or significant arrhythmia were excluded. Informed consent was obtained after CCTA or after ICA, and 258 participants were enrolled between October 2021 and November 2022.

### CT analysis

The aorta and coronary arteries were extracted from the best image quality phase available using a combination of automated algorithms and human quality review. All coronary arteries visible on CCTA were analysed for total plaque volume (TPV), vessel volume, lumen volume, calcified plaque (CP), non-calcified plaque (NCP), and low-attenuation plaque (LAP) volumes. AI-QCPA (HeartFlow, Mountain View, CA) was used to produce a patient-specific 3D model of the arterial lumen and outer wall from CCTA to quantify and characterize plaque. Deep learning algorithms, trained on millions of training samples from annotated CCTA images, were used to segment the lumen and outer vessel wall. Certified CT analysts then performed quality-checks on segmentations and modified lumen boundaries in each case where necessary using a prescribed predefined process and a custom workstation for inspecting the AI algorithm results overlaid on the CCTA image data. Once the lumen and outer wall were segmented, plaque volumes were quantified, and plaque was characterized using automatic thresholding based on Hounsfield units (LAP, between −30 and 30 HU, and NCP ≥30 HU or <CP threshold). The CP was derived with adaptive thresholding based on lumen contrast, wherein threshold for CP was set to the maximum of 350 HU or the average lumen intensity plus 1 SD of the lumen intensities. The supplementary material contains a detailed description of the methodology, training, and validation of the AI-QCPA tool set based on the PRIME checklist.^8^

Cross-sectional analysis provided lumen area, vessel area, plaque area (vessel area − lumen area), and its components (CP and NCP area). Vessel, lumen, and plaque volumes were calculated using numerical integration with Simpson’s rule. TPV was calculated as the sum of volumes of the individual plaque components. NCP volume was calculated by subtracting CP volume from TPV. The presence or absence of LAP on a per-lesion basis was analysed using a range of prespecified thresholds for LAP presence by IVUS at 2, 4, and 8 mm^3^ to construct receiver operating characteristic (ROC) curves and determine sensitivity, specificity, PPV, NPV, and accuracy of AI-QCPA for LAP.

### IVUS acquisition

IVUS was performed using one of the following systems: OptiCross 60 MHz (Boston Scientific, Fremont, CA), Refinity 45 MHz, Eagle Eye 20 MHz (Philips, Rancho Cordova, CA), AltaView 60 MHz (Terumo, Tokyo, Japan), or Dualpro 50 MHz (InfraReDx/Nipro, Bedford, MA). After administration of intracoronary nitroglycerin, IVUS imaging was performed with motorized pullback at 0.5 mm/s to include 6–10 cm of the coronary artery to the aorto-ostium. IVUS images were archived as DICOM and sent to an independent IVUS core laboratory (Cardiovascular Research Foundation, New York, NY) for quantitative and qualitative analyses with validated planimetry software (ImageJ, v 1.53).^9^

### IVUS analysis

**Figure 1:**
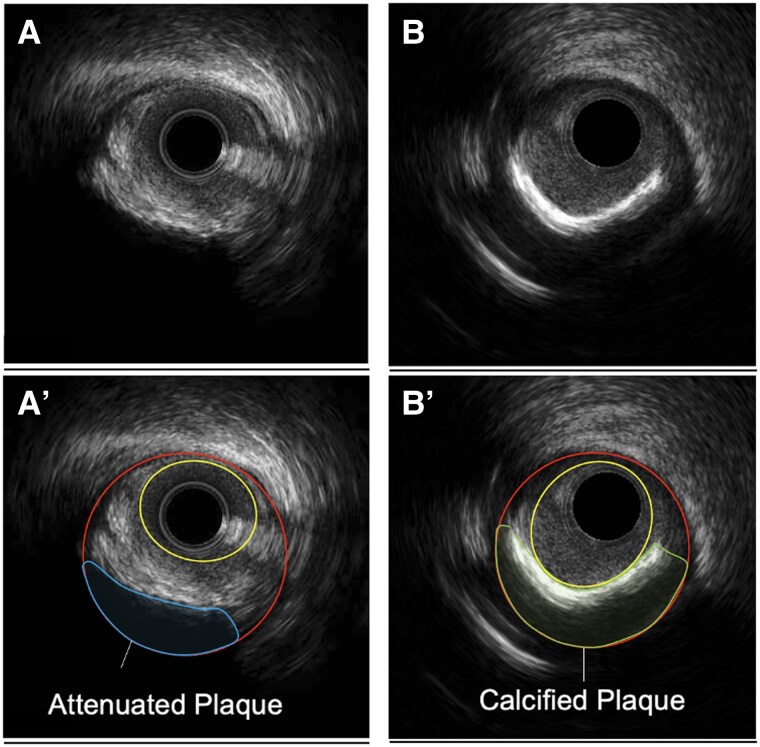
Example of annotated IVUS frames showing lumen area, outer wall area, attenuated plaque area, and calcified plaque area. Panels *A'* and *B'* are identical to Panels *A* and *B*, but they include additional explanatory information in the form of annotations. The area enclosed by the yellow circle represents the lumen. The red area demarcates the outer wall of the blood vessel. The blue area denotes attenuated plaque. The green area points out calcified plaque.

Quantitative analysis included measurement at 1 mm increments of the vessel (corresponding to external elastic membrane) and lumen cross-sectional areas. Plaque plus media area was calculated as vessel minus lumen area. Once a complete set of vessel and lumen area measurements was obtained, lumen and vessel volume were calculated by Simpson’s rule. Qualitative analysis included identification of calcification and attenuated plaque^10^ (corresponding to lipidic plaque). Calcification was defined as areas brighter than the reference adventitia with acoustic shadowing with or without reverberation. Attenuated plaque was defined by the absence of the ultrasound signal behind plaque that was either hypoechoic or isoechoic to the reference adventitia but contained no bright calcium. Although ultrasound is unable to evaluate the tissue characteristics behind calcium or attenuated plaque, the entire thickness of plaque was assumed to be same as surface plaque (*Figure 1*).

### Co-registration process

**Figure 2:**
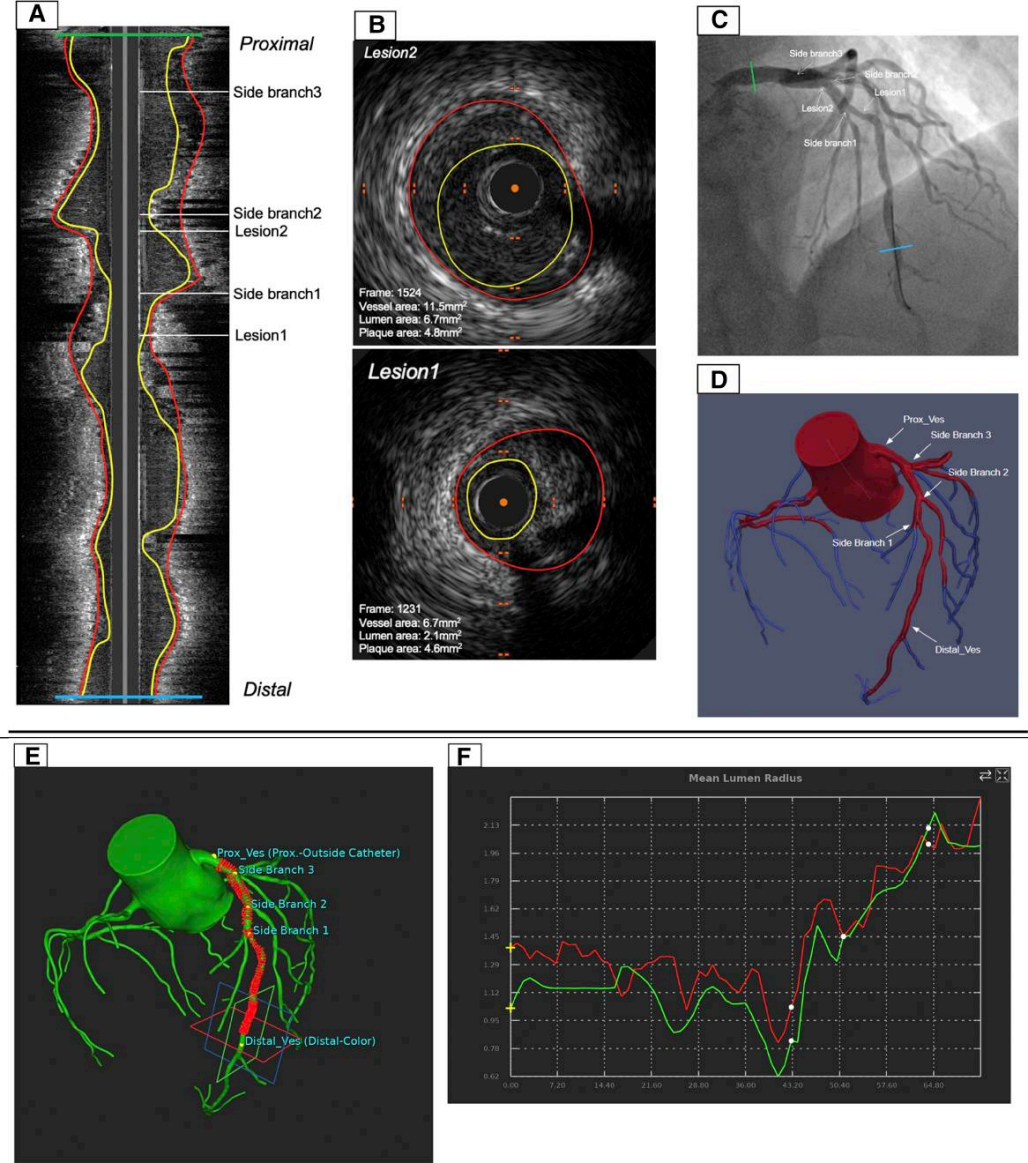
Example of co-registration between IVUS, angio, and CCTA. The core lab-annotated various features on IVUS slices, including the proximal and distal ends of pullback, bifurcations, and lesion locations (*A*). The minimal lumen area (MLA) within the lesion was marked (*B*), and these annotations were co-registered with angiography images (*C*). These same features on the IVUS were also co-registered with a CCTA-based 3D model (*D*). Using ImFusion software (*E*), the IVUS and CCTA lumen area profiles were aligned (red line for IVUS, green line for CCTA). Post-alignment, the CCTA 3D locations for the proximal and distal ends of lesions were outputted for final plaque volume extraction.

CCTA-based AI-QCPA analyses were carried out in the lesions and segments defined by the IVUS core lab. A lesion was defined as any segment with plaque burden ≥40% and ≥2 mm in length. The start and end locations of each pullback region and each lesion were denoted on corresponding 3D locations on the CCTA images using the IVUS core lab output. A co-registration tool was developed for this purpose (ImFusion Suite, ImFusion GmbH, Munich, Germany). The inputs to this tool were co-registration points for the pullback ranges, bifurcations, and lumen segmentations per IVUS slice. A trained expert not involved in algorithm development co-registered the 1D lumen profiles from the IVUS and CCTA lumen segmentations. Bifurcation and pullback start and endpoints were used as anchor points for the co-registration. Computation of plaque volumes was limited to the co-registered lesion and pullback ranges from the core lab and conducted automatically. An example of the co-registration process is shown in *Figure 2*.

### Statistical analysis

A sample size of 223 subjects was needed to provide 90% power to detect agreement if mean plaque volume was 8 mm^3^ and SD 12 mm^3^. All continuous variables are expressed as mean ± SD. Categorical variables are presented as number (percent). Agreement between AI-QCPA and IVUS was evaluated with the use of intra-class correlation coefficients along with Bland–Altman analyses. Pearson correlations were calculated to compare plaque volumes between AI-QCPA and IVUS. Paired samples *t*-test was used to verify the differences between paired observations. *P*-values < 0.05 were considered statistically significant. Statistical analyses were performed with the use of SAS software version 9.4 (SAS Institute, Cary, North Carolina).

## Results

**Figure 3:**
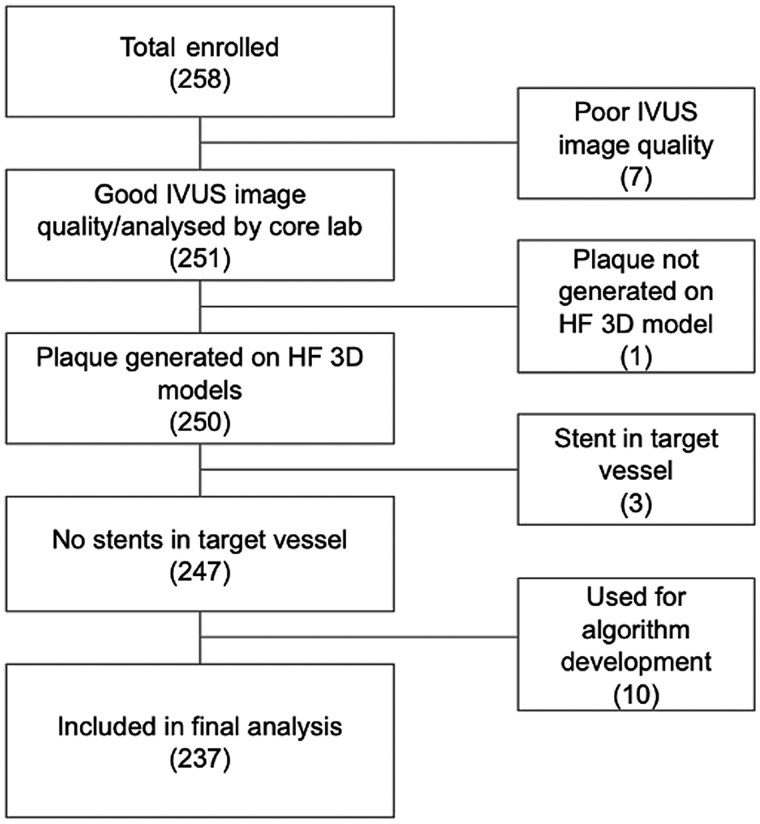
Consort diagram of enrolled patients included in statistical analysis. Out of 258 enrolled patients, 21 were excluded from the final analysis due to reasons including poor IVUS image quality (7 patients), inability to generate plaque on the CCTA-based 3D model (1 patient), presence of non-analysable stent in the target vessel by AI-QCPA (3 patients), and usage of data for AI-QCPA algorithm development (10 patients). The final statistical analysis was conducted with 237 patients, accounting for 432 lesions and 245 IVUS pullbacks.

**Figure 4:**
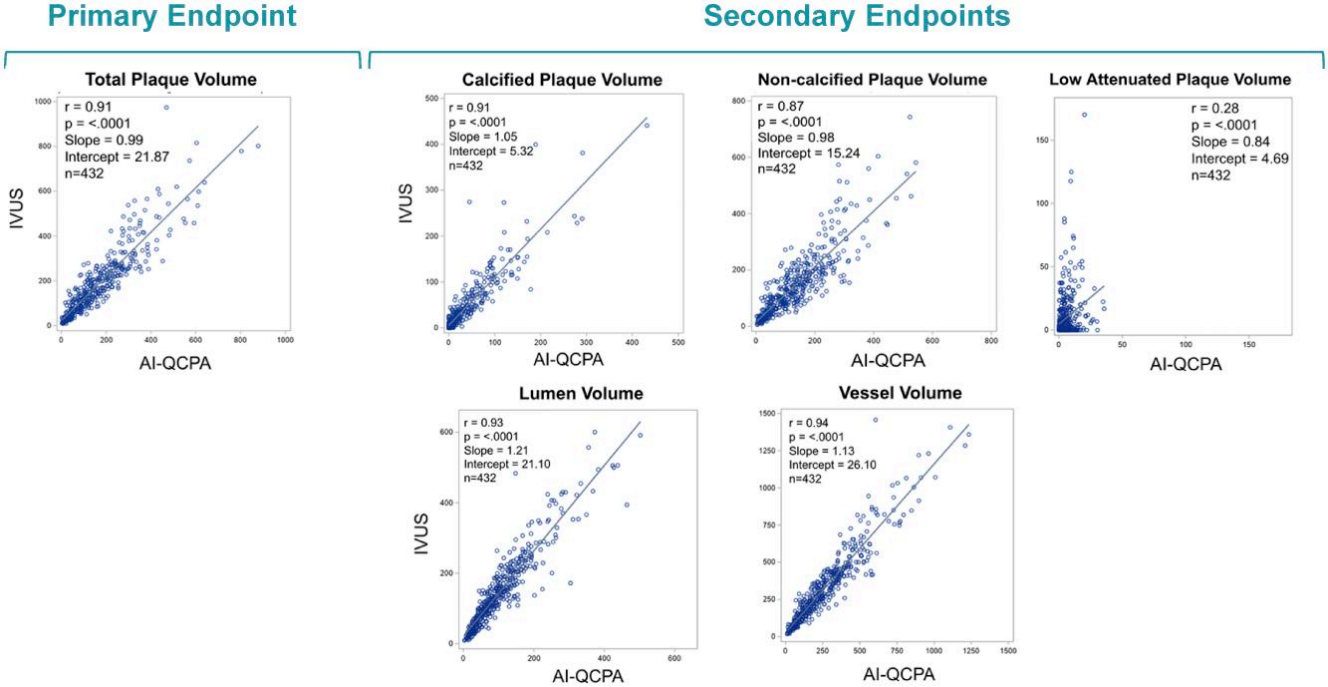
Scatterplots with regression line, slope, and intercept showing correlation between AI-QCPA and IVUS on a per-lesion basis. The figure displays scatterplots with regression lines illustrating the correlation between AI-QCPA and IVUS measurements. Each part is based on a per-lesion basis (*n* = 432). The primary endpoint includes TPV with a Pearson’s correlation of 0.91. The secondary endpoints include CP volume with a Pearson’s correlation of 0.91, NCP volume with a Pearson’s correlation of 0.87, LAP volume with a Pearson’s correlation of 0.28, lumen volume with a Pearson’s correlation of 0.93, and vessel volume with a Pearson’s correlation of 0.94.

Of the 258 patients enrolled, 237 were included in the analysis (*Figure 3*). Demographics and baseline characteristics for 237 patients are shown in *Table 1*, which included 245 IVUS pullbacks and 432 lesions. Of these lesions, 275 (64%) were in the left anterior descending coronary artery (LAD), 88 (20%) in the right coronary artery (RCA), and 69 (16%) in the left circumflex artery (LCX). Mean length of lesions was 24.5 ± 16.6 mm. Absolute plaque volume and percentage of each plaque components are shown in *Table 2*. The relationship between AI-QCPA and IVUS plaque volumes is illustrated through scatterplots with regression lines, slopes, and intercepts (*Figure 4*).

**Table 1:** Demographics and baseline characteristics of study population (*n* = 237)

Mean age in years	67.5 (±9.8)
**Birth sex**	
Men	176 (74.3%)
Women	61 (25.7%)
**Body mass index (kg/m^2^)**	29.1 (±5.9)
**Race**	
White	157 (66.2%)
Asian	63 (26.6%)
Japanese	61 (96.8%)
Asian Indian	2 (3.2%)
Black or African American	9 (3.8%)
Other/unknown	8 (3.4%)
**Ethnicity**	
Not Hispanic, Latino/a, or Spanish origin	227 (95.8%)
Hispanic, Latino/a, or Spanish origin	9 (3.8%)
Other/unknown	1 (0.4%)
**Medical history**	
Angina within 45 days of CCTA	149 (62.9%)
Congestive heart failure	12 (5.1%)
Diabetes	78 (32.9%)
Hypertension	187 (78.9%)
Hyperlipidaemia	203 (85.7%)
Former smoker	94 (39.7%)
Current smoker	31 (13.1%)
Nicotine use 24 h prior to CCTA	7 (3%)
Family history of premature atherosclerotic disease	56 (23.6%)
Stroke	22 (9.3%)
Transient ischaemic attack	6 (2.5%)
Peripheral vascular disease	18 (7.6%)
**Other cardiovascular investigations within 90 days of CCTA**	
Invasive coronary angiography (post-CCTA)	27 (11.4%)
Exercise tolerance test	3 (1.2%)
Stress echocardiogram test	2 (0.8%)
Nuclear myocardial perfusion scan test	30 (12.7%)
Cardiac MRI test	1 (0.4%)

**Table 2:** Plaque components measured by IVUS and CCTA (*n* = 432 lesions)

	IVUS by experts	CCTA by AI-QCPA
TPV (mm^3^)	155.3 (79.2, 254.6)	132.1 (65.5, 237.6)
CP volume (mm^3^)	15.4 (1.8, 48.5)	10.1 (0.3, 42.5)
Percent CP volume^a^ (%)	12.0 (1.9, 27.1)	8.8 (0.5, 22.7)
NCP volume (mm^3^)	123.6 (67.1, 199.2)	118.5 (55.3, 187.5)
Percent NCP volume^a^ (%)	88.0 (72.9, 98.1)	91.2 (77.3, 99.5)
LAP volume (mm^3^)	1.7 (0, 10.9)	3.3 (1.0, 7.3)
Percent LAP volume^a^ (%)	1.1 (0, 6.5)	2.4 (1.1, 4.5)

Data are shown as median (first quartile, third quartile).

CP, calcified plaque; LAP, low-attenuation plaque; NCP, non-calcified plaque; TPV, total plaque volume.

^a^Percent volume is defined as the ratio compared with the TPV.

A Pearson’s correlation analysis conducted across all vessels on a per-lesion basis for vessel, lumen, and plaque volumes demonstrated significant correlation for all—TPV (0.91, *P* < 0.0001), vessel volume (0.94, *P* < 0.0001), lumen volume (0.93, *P* < 0.0001), CP volume (0.91, *P* < 0.0001), NCP volume (0.87, *P* < 0.0001), and LAP volume (0.28, *P* < 0.0001). The slopes of the Pearson’s correlation approximated 1 for all measures except 0.84 for LAP, and the Y intercepts were low (*Figure 4*). Strong correlation was also found for each specific vessel distribution: 0.91 for the LAD (*n* = 275), 0.92 for the RCA (*n* = 88), and 0.93 for the LCX (*n* = 69) (correlation analysis stratified by scan acquisition tube potential and CT scan platform are included in the Supplementary appendix).

**Figure 5:**
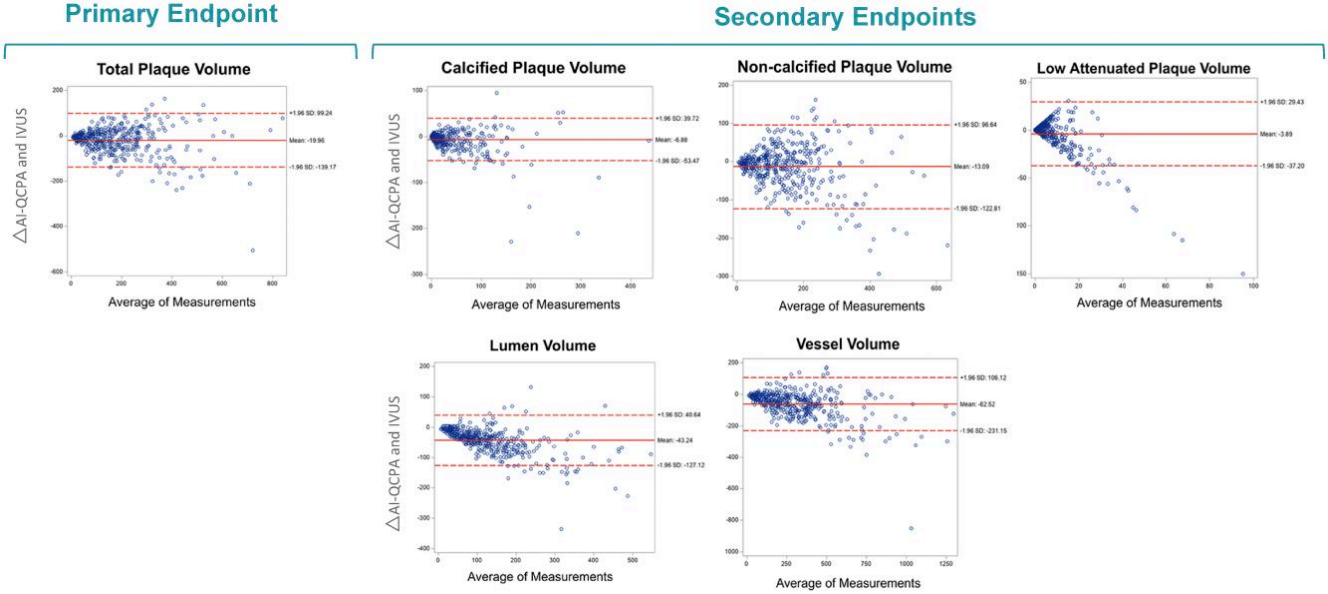
Bland–Altman plots with mean differences and limits of agreements comparing AI-QCPA and IVUS on a per-lesion basis. The figure presents Bland–Altman plots illustrating the mean differences between AI-QCPA and IVUS on a per-lesion basis. Limits of agreement were calculated using a 95% confidence interval (mean difference ± 1.96 times SD). The primary endpoint includes TPV with a mean difference of −19.6. The secondary endpoints include CP with a mean difference of −6.88, NCP with a mean difference of −13.09, LAP with a mean difference of −3.89, vessel volume with a mean difference of −62.52, and lumen volume with a mean difference of −43.24.

Using Bland–Altman analysis (*Figure 5*), there was slight underestimation of plaque volumes on CCTA compared with IVUS with mean differences of −19.96 mm^3^ for TPV, −6.88 mm^3^ for CP, −13.09 mm^3^ for NCP, and −3.89 mm^3^ for LAP. The agreement limits were calculated using a 95% confidence interval, established as the mean difference ± 1.96 times the SD (Bland–Altman analysis also provided by acquisition tube potential and CT scan platform are included in the Supplementary appendix).

**Figure 6:**
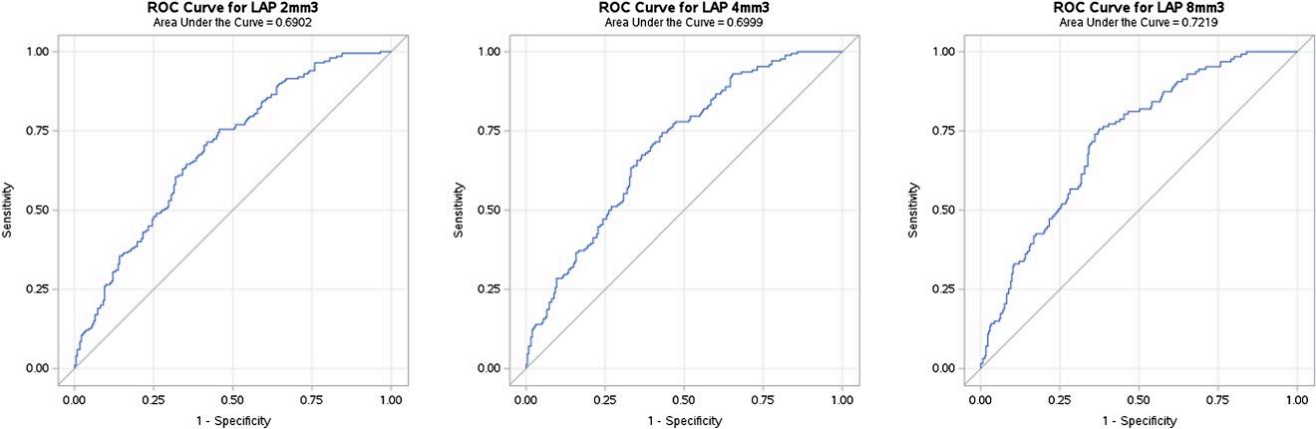
ROC curve for LAP. ROC curve for LAP analysis: A binary comparative analysis was conducted to assess the presence of LAP as detected by IVUS vs. CCTA. Three different thresholds for LAP presence (left, 2 mm³; middle, 4 mm³; right, 8 mm³). Threshold of 8 mm³ demonstrated an AUC of 0.72, while threshold of 4 mm³ led to AUC of 0.70, and threshold of 2 mm³ resulted in AUC of 0.69.

IVUS-detected LAP in 251 lesions and the median value of LAP was 8.1 mm^3^. Based on the median value of LAP, we specified 8.0 mm^3^ as LAP threshold. The threshold of 2 and 4 were defined arbitrarily as a comparison of 8.0 mm^3^. ROC analysis of LAP presence or absence on a per-lesion basis (*Figure 6*) yielded AUC values of 0.69, 0.7, and 0.72 for IVUS thresholds of 2, 4, and 8 mm^3^, respectively. *Table 3* reports sensitivity, specificity, positive predictive value (PPV), and negative predictive value (NPV) for IVUS thresholds of 2, 4, and 8 mm^3^.

**Table 3:** LAP sensitivity, specificity, accuracy, and AUC for various thresholds

LAP volume threshold	Sensitivity (%)	Specificity (%)	Accuracy (%)	AUC
2 mm^3^	77	50	62	0.69
4 mm^3^	60	67	64	0.70
8 mm^3^	39	84	71	0.72

## Discussion

This large international multicentre study demonstrated a significant correlation of AI-based CCTA analysis with core laboratory-adjudicated IVUS measurements across all plaque volumes. The significance of these findings is underscored by the overwhelming evidence supporting plaque volumes and composition in determining patient risk and as markers for monitoring disease progression. The potential of CCTA to quantify and describe atherosclerosis, refine risk prediction, and guide medical management over time is reliant on the precision of the method, highlighting the importance of our findings.

The study results demonstrate that AI-derived CCTA plaque volumes correlate significantly with IVUS for TPV, CP volume, and NCP volume and that AI-QCPA could serve as a viable non-invasive surrogate to invasive methods of assessment of plaque burden. The concordance between AI-QCPA and IVUS segmentation was excellent, with correlation coefficients and linear regression slopes nearing 1.0 and Y intercepts nearing zero. Furthermore, the correlation was high for the subsets of samples derived for all LAD, LCX, and RCA territories. This finding has considerable clinical relevance, as the association between TPV and patient risk is being increasingly acknowledged, in addition to the evolving evidence for the transition from NCP to CP through medical management, particularly with statins. Importantly, our study includes imaging from many centres across all commonly used CT scan platforms and image reconstruction algorithms with varied tube potentials supporting the generalizability of our findings.

Although the correlation between AI-QCPA and IVUS for LAP was not near perfect as that for TPV, and CP and NCP volumes, it was not unexpected due to the inherent differences in definitions, threshold settings, and methodology for identifying LAP between IVUS and CCTA. Although capable of defining plaque necrotic core, IVUS struggles to discern lipid-rich regions deeper within the vessel wall. Conversely, LAP evaluation using a Hounsfield unit threshold by CCTA has inherent limitations at the outer limits of the coronary vessel wall and is influenced by a narrow range of Hounsfield units and varied tube potential. We anticipate that future machine learning approaches may yield improved correlations for quantifying LAP with IVUS.

The data from our current analysis form the foundation to facilitate the thoughtful incorporation of AI-QCPA. Like any diagnostic tool, stepwise evaluation is necessary before clinical integration. A hierarchical approach, such as the one proposed in REFERAL (Long-term Clinical Outcomes in Patients With FFR Guided-Deferred Coronary Lesions, Assessed by IVUS Analysis) registry (NCT04068779), underlines the need to establish reproducibility and accuracy before clinical integration. Moreover, recent studies such as the EMERALD I provides evidence on the relationship between physiology and atherosclerosis, which may prove invaluable in future clinical applications of AI-QCPA. The recently published nomographic values of plaque burden using the AI-QCPA tool have facilitated the stratification of plaque burden by age and sex, enhancing our understanding of how to inform clinical practice. The logical next step will be to prospectively examine the impact of atherosclerosis burden on clinical decision-making, downstream cardiovascular outcomes, and costs.

Our analysis does have limitations; IVUS is unable to evaluate tissue characteristics behind calcium or attenuated plaque. Therefore, the complete thickness of plaque is assumed to be the same as surface plaque, which could lead to an overestimation of IVUS analysed attenuated or calcified plaque volumes. Also, the current study only assessed quantitative accuracy, and clinical outcomes were not obtained. Finally, while our study included a broad range of CT scan platforms and varied acquisition parameters, our analysis was not sufficiently powered to explore sub-analyses of agreement of AI-QCPA stratified by acquisition parameters, but we have provided correlation and Bland–Altman analysis stratified by CT scan platform and tube potential in the Supplementary appendix. We unfortunately do not have consistent data on CT scan reconstruction algorithm or level of iterative reconstruction used across the CT scans.

## Conclusion

This prospective, blinded, core lab-adjudicated international, multicentre study of an AI-based automated CCTA plaque characterization demonstrated excellent accuracy and precision with current reference standard of IVUS-verified lumen, plaque volume, and lesion morphology. AI-QCPA is a robust non-invasive tool for the quantitation and characterization of coronary atherosclerosis. The results of this study further support the position of CCTA as a viable non-invasive diagnostic strategy for a comprehensive evaluation of coronary artery disease.

## Supplementary data

Supplementary data are available at *European Heart Journal - Cardiovascular Imaging* online.

## Supplementary Material

jeae115_Supplementary_Data

jeae115_Supplementary_Material

jeae115_Supplementary_Appendix

jeae115_Supplementary_Data

## Data Availability

The data underlying this article will be shared upon reasonable request to the corresponding author.
