# Supplementary material for: Prospective deep learning–based quantitative assessment of coronary plaque by computed tomography angiography compared with intravascular ultrasound: the REVEALPLAQUE study
Source: Eur Heart J Cardiovasc Imaging. 2024 May 3;25(9):1287–95. doi: 10.1093/ehjci/jeae115 (PMC11346368; doi:10.1093/ehjci/jeae115)
Supplement: jeae115_Supplementary_Data [file jeae115_supplementary_data.docx]

**Appendix**

**Table. Computed Tomography Parameters**

| **Manufacturer** | **Count (%)** |
| --- | --- |
| Siemens | 112/237 (47.3%) |
| GE Medical Systems | 82/237 (34.6%) |
| Canon Medical Systems | 40/237 (16.9%) |
| Philips | 3/237 (1.3%) |
| **Model** |  |
| SIEMENS SOMATOM Force | 71/237 (30.0%) |
| SIEMENS SOMATOM Definition AS+ | 32/237 (13.5%) |
| SIEMENS SOMATOM Definition Flash | 9/237 (3.8%) |
| GE Revolution CT | 62/237 (26.2%) |
| GE Revolution EVO | 12/237 (5.1%) |
| GE LightSpeed VCT | 4/237 (1.7%) |
| GE Revolution HD | 2/237 (0.8%) |
| GE Discovery CT750 HD | 1/237 (0.4%) |
| GE Revolution Apex | 1/237 (0.4%) |
| Canon Aquilion ONE | 40/237 (16.9%) |
| Philips iCT 256 | 3/237 (1.3%) |
| **kVP** |  |
| 70 | 1/237 (0.4%) |
| 80 | 1/237 (0.4%) |
| 90 | 6/237 (2.5%) |
| 100 | 58/237 (24.5%) |
| 110 | 16/237 (6.8%) |
| 120 | 145/237 (61.2%) |
| 130 | 3/237 (1.3%) |
| 140 | 7/237 (3.0%) |
| **Slice Thickness (mm)** |  |
| 0.5 | 40/237 (16.9%) |
| 0.6 | 67/237 (28.3%) |
| 0.625 | 82/237 (34.6%) |
| 0.67 | 2/237 (0.8%) |
| 0.75 | 45/237 (19.0%) |
| 0.9 | 1/237 (0.4%) |
| **Reconstruction Algorithm** |  |
| Iterative (ADMIRE OR SAFIRE) | 80/236 (33.9%) |
| AiDR 3D | 39/236 (16.5%) |
| AR | 34/236 (14.4%) |
| FBP | 32/236 (13.6%) |
| DLIR | 30/236 (12.7%) |
| SSXX:Slice | 18/236 (7.6%) |
| iDose | 3/236 (1.3%) |

ADMIRE = advanced modeled iterative reconstruction; AiDR = adaptive iterative dose reduction; AR = analytical reconstruction; OLIR = deep leaning image reconstruction; FBP = filtered back projection; SAFIRE = sinogram affirmed iterative reconstruction
