# Supplementary material for: Prospective deep learning–based quantitative assessment of coronary plaque by computed tomography angiography compared with intravascular ultrasound: the REVEALPLAQUE study
Source: Eur Heart J Cardiovasc Imaging. 2024 May 3;25(9):1287–95. doi: 10.1093/ehjci/jeae115 (PMC11346368; doi:10.1093/ehjci/jeae115)
Supplement: jeae115_Supplementary_Material [file jeae115_supplementary_material.docx]

*Narula, et al., Quantitative Assessment of Deep Learning-based Coronary Plaque by CT Angiography Prospectively Compared with Intravascular Ultrasound. A pRospEctiVe, multicEnter study to AnaLyze PLAQUE using CCTA: REVEALPLAQUE.*

**Supplementary material**

Following is a detailed description of the methodology, training, and validation of the Artificial Intelligence-Enabled Quantitative Coronary Plaque Analysis (AI-QCPA) tool used in the paper based on the PRIME checklist^1^.

**Designing the Study Plan**

1. Describe the need for the application of machine learning to the dataset.
   - *Clinically, coronary plaque characterization may inform patient-specific risk stratification and management strategies. While visual assessment of coronary plaque appearance is considered an important component of a contemporary CCTA report, according to the CAD-RADS 2.0 reporting guidelines, manual plaque segmentation has proven challenging by virtue of time requirements and limited inter- and intra-observer reproducibility. An Artificial Intelligence-Enabled Quantitative Coronary Plaque Analysis (AI-QCPA) tool could greatly facilitate assessment of coronary atherosclerosis from CCTA data.*
2. Describe the objectives of the machine learning analysis.
   - *Automatic segmentation of the lumen and the outer wall of coronary arteries in CCTA.*
3. Define the study plan.
   - *Evaluate the level of agreement between automated noninvasive CCTA quantification and characterization of coronary plaque volumes (AI-QCPA), and Intravascular Ultrasound (IVUS) data.*
4. Describe the summary statistics of baseline data.
   - *The lumen segmentation algorithm was trained on 6694 CCTA cases randomly selected from routine clinical use cases of HeartFlow’s FFRct product. The lumen segmentation for these cases were segmented with an earlier version of HeartFlow’s deep learning segmentation model and reviewed and corrected by trained analysts for use in the FFRct product. The outer wall segmentation algorithm was trained on two datasets: a set of 818 CCTA cases annotated with the AutoPlaque software (Cedars-Sinai, Los Angeles, CA) by trained analysts at Cedars-Sinai and 1800 additional CCTA cases annotated by trained analysts with proprietary software developed for the HeartFlow analysis (HeartFlow Inc., Mountain View, CA).* *The 1800 additional cases were randomly selected from routine clinical use of HeartFlow’s FFRct product, i.e. patients with at least one diameter stenosis greater than 30% narrowing. It is therefore expected that training on these cases will produce a model that generalizes well to all symptomatic patients with at least one diameter stenosis greater than 30% narrowing.
     Exact patient statistics of the cases used to train the lumen boundary and outer wall were not known to the HeartFlow development team, but it is expected that the ADVANCE registry statistics^2^ closely match HeartFlow’s commercial population and thereby also these training cases.*
5. Describe the overall steps of the machine learning workflow.
   - *The evaluated method relied on a combination of AI algorithms and a human quality review process to produce a patient-specific 3D model of the arterial lumen and outer wall from CCTA. The aorta and coronary arteries were extracted from the best image quality phase available using a combination of automated algorithms and human quality review.
     Deep learning algorithms were used to segment the lumen and outer vessel wall. For each vessel in a case, image data was converted to a stack of 2D image slices extracted cross-sectionally to the centerline. For each cross-section, regression targets for the deep learning algorithms were defined as distances along equiangular rays from the centerline to the outer wall boundary. After inference, a surface reconstruction algorithm built a water-tight mesh from the regressed distances around all the vessels of a case. Certified CT analysts then performed quality-checks on segmentations, and lumen boundaries were modified if necessary using a prescribed predefined process and a custom workstation for inspecting the AI-algorithm results overlaid on the CCTA image data. Once the lumen and outer wall were segmented, plaque volumes were quantified and plaque characterized using thresholding techniques described in the main manuscript.*
   - *Since the automated lumen segmentation is corrected by analysts and the outer wall segmentation is used without corrections, the remainder of this document will focus on the outer wall segmentation model.*

**Data Standardization, Feature Engineering, and Learning**

1. Describe how the data were processed in order to make it clean, uniform, and consistent.
   - *The AutoPlaque cases contained annotations of the outer wall in diseased sections and the additional cases included algorithmically modified lumen segmentations in healthy sections. In the healthy sections, the outer wall was modeled as the lumen segmentation used for the HeartFlow analysis dilated by 20% of the local diameter. All annotations were stored as 3D volumetric implicit surfaces. For each vessel in the case, the image data was converted to a stack of 2D 61 x 61 image slices with 0.25 x 0.25 mm^2^ pixel size extracted cross-sectionally to the centerline. For each cross-section, regression targets were defined as 32 distances along equiangular rays from the centerline to the outer wall boundary.*
2. Describe whether variables were normalized and if so, how this was done.
   - *The image data was normalized with three different approaches leading to three separate input channels. The first normalization technique was a standard normalization where the mean and standard deviation were computed from the intensities of the stack of 2D slices for improved robustness against local artifacts. The second normalization technique regressed the intensity profile along the centerline using robust Gaussian kernel regression. Each cross-sectional slice was then divided by the regressed intensity value of the associated cross-section. The third technique described an affine mapping from image intensities to a similar intensity range as the first two normalizations. For this, the typical intensity of lumen (HU= 500) was subtracted from the given intensity value and divided by 1000, the difference of a typical intensity value for calcium (HU=1000) and water (HU=0).*
3. Provide details on the fraction of missing values (if any) and imputation methods.
   - *At bifurcations, when rays intersected a branch vessel, target distances were determined to be missing. These missing values were imputed by robust Gaussian kernel regression.*
4. Describe any feature selection process applied.
   - *No feature selection was performed.*
5. Identify and describe the process to handle outliers, if any.
   - *Nothing was done specifically for outlier target distances.*
6. Describe whether class imbalance existed, and which method was applied to deal with it.
   - *Since this is a regression task, no class imbalance exists. However, the model was trained while making sure to select at every stage 50% from both the AutoPlaque cases and the additional cases to ensure the resulting model was not biased to either of the two datasets.*

**Selection of Machine Learning Models**

1. Explicitly define the goal of the analysis e.g., regression, classification, clustering
   - *A deep learning model was trained to predict the target distances given the normalized image data using a regularized mean square error loss function, the canonical link function for regression problems.
     The input of the network was provided in the ring representation^3^, multi-channel 3D image sub-volumes of size 3x32x45x45 taken from the cross-sectional image stacks. The four dimensions capture the normalization channels, rotational direction, longitudinal direction, and the direction of a ray within the cross-sectional slice.*
2. Identify the proper learning method used (e.g. supervised, reinforcement learning, etc) to address the problem
   - *The model is trained fully supervised*
3. Provide explicit details on the use of simpler, complex, or ensemble models
   - *Several different models were tested (with varying number of layers, number of kernels per layer, kernel size, activation functions, use of drop out, use of skip connections, etc.) and the model with the best performance on the validation set was selected. Ensemble models were not tested.*
4. Provide the comparison of complex models against simpler models if possible
   - *The comparison results were not available at the time of writing this manuscript.*
5. Define ensemble methods, if used
   - *No ensemble methods were used*
6. Provide details on whether the model is interpretable
   - *No specific effort was made to make the model interpretable.*

**Model Assessment**

1. Provide a clear description of data used for training, validation, and testing
   1. *The hyper-parameters of the outer wall segmentation architecture were optimized on a separate validation set of 1000 subvolumes from 66 AutoPlaque cases and another 1000 subvolumes from 200 additional cases using the mean square surface error as the objective function. Models of several levels of complexity were tested and the model with the best performance on the validation set was selected.*
2. Describe how the model parameters were optimized (e.g., optimization technique, number of model parameters, etc.)
   1. *The hyper-parameter optimization of the outer wall segmentation model resulted in a network that was fully convolutional and included leaky rectified linear units as activation functions. The network contained eight 3x3 convolutional layers with respectively 32 kernels and two fully connected layers with respectively 64 and 128 kernels. For hyper-parameter optimization and training, 4 million training samples were randomly sampled from the cross-sectional stacks.
      The model was optimized using the stochastic gradient descent optimizer, learning rate of 1e-2, momentum 0.9, exponential Learning rate schedule gamma of 0.999975, gradient clipping of 5, drop out, group norm normalization with 4 groups and batch size of 50. During training, several forms of image intensity augmentation (scaling, additive Gaussian noise) were applied to increase the robustness of the model.*

**Model Evaluation**

1. Provide the metric(s) used to evaluate the performance of the model
   1. *The mean square error was used as an objective function during model selection.*
2. Define the prevalence of disease and the choice of the scoring rule used
   1. *The training data was composed such that 50% of the training samples came from diseased sections and 50% from healthy sections.*
3. Report any methods used to balance the numbers of subjects in each class
   1. *This is not applicable since a regression model was trained*
4. Discuss the risk associated to misclassification
   1. *It is acknowledged that the deep learning model can make regression mistakes on unseen data and this needs to be taken into account during clinical application. The primary purpose of this study was to validate the deep learning plaque quantification methods against ground-truth invasive data.*

**Best Practices for Model Replicability**

1. Consider sharing code or scripts on a public repository with appropriate copyright protection steps for further development and non-commercial use
   1. *HeartFlow considered this, but decided against this since these quantitative plaque algorithms are proprietary.*
2. Release a data dictionary with appropriate explanation of the variables
   1. *Not applicable. No explicit variables were defined since the model was trained with raw image data as input.*
3. Document the version of all software and external libraries used
   1. *The model was trained with PyTorch version pytorch 1.13.1, pytorch-cuda 11.6, ray 2.3.0, scipy 1.81, einops 0.61*

**Reporting Limitations, Biases and Alternatives**

1. Identify and report the relevant model assumptions and findings
   1. *The model was trained on a significant amount of training data representing a wide variety of patients and disease patterns that is expected to match well the target population. Moreover, during training, data augmentation was used to increase generalizability and robustness. However, it can still happen that the model is presented with a section of coronary CCTA data that is not represented well in the training distribution. In this case, it is possible that the model predicts incorrect distances.*
2. If well performing models were tested on a hold-out validation dataset, detail the data of that validation set with the same rigor as that of training dataset
   1. *See above in the “Model Assessment” section.*

**REFERENCES:**

1. Sengupta PP, Shrestha S, Berthon B, Messas E, Donal E, Tison GH, Min JK, D'hooge J, Voigt JU, Dudley J, Verjans JW, Shameer K, Johnson K, Lovstakken L, Tabassian M, Piccirilli M, Pernot M, Yanamala N, Duchateau N, Kagiyama N, Bernard O, Slomka P, Deo R, Arnaout R. Proposed Requirements for Cardiovascular Imaging-Related Machine Learning Evaluation (PRIME): A Checklist: Reviewed by the American College of Cardiology Healthcare Innovation Council. JACC Cardiovasc Imaging. 2020 Sep;13(9):2017-2035. doi: 10.1016/j.jcmg.2020.07.015. PMID: 32912474; PMCID: PMC7953597.
2. Fairbairn TA, Nieman K, Akasaka T, Nørgaard BL, Berman DS, Raff G, Hurwitz-Koweek LM, Pontone G, Kawasaki T, Sand NP, Jensen JM, Amano T, Poon M, Øvrehus K, Sonck J, Rabbat M, Mullen S, De Bruyne B, Rogers C, Matsuo H, Bax JJ, Leipsic J, Patel MR. Real-world clinical utility and impact on clinical decision-making of coronary computed tomography angiography-derived fractional flow reserve: lessons from the ADVANCE Registry. Eur Heart J. 2018 Nov 1;39(41):3701-3711. doi: 10.1093/eurheartj/ehy530. PMID: 30165613; PMCID: PMC6215963.
3. Petersen, K., Schaap M., Lesage, D., Grady, L. DeepLumen: Fast and Accurate Segmentation of Coronary Arteries for Improved Cardiovascular Care. Paper presented at: NVIDIA GTC (GPU Technology Conference); May 10, 2017; San Jose, CA.
