# Supplementary material for: Prospective deep learning–based quantitative assessment of coronary plaque by computed tomography angiography compared with intravascular ultrasound: the REVEALPLAQUE study
Source: Eur Heart J Cardiovasc Imaging. 2024 May 3;25(9):1287–95. doi: 10.1093/ehjci/jeae115 (PMC11346368; doi:10.1093/ehjci/jeae115)

Correlation Tables

Plaque Correlations by Scanner Platform/Type

Patients	Siemens SOMATOM Force (n=71)						GE Revolution CT (n=62)						Canon Aquilion ONE (n=40)					
	Per-Vessel (n=72)			Per-Lesion (n=125)			Per-Vessel (n=67)			Per-Lesion (n=112)			Per-Vessel (n=41)			Per-Lesion (n=72)		
Variables	Vessel Pearson	Vessel Spearman	ICC Vessel	Lesion Pearson	Lesion Spearman	ICC Lesion	Vessel Pearson	Vessel Spearman	ICC Vessel	Lesion Pearson	Lesion Spearman	ICC Lesion	Vessel Pearson	Vessel Spearman	ICC Vessel	Lesion Pearson	Lesion Spearman	ICC Lesion
TPV	0.8700 [0.7995-0.9169]	0.8558 [0.7785-0.9075]	0.9146 [0.86-0.95]	0.9210 [0.8892-0.9439]	0.9428 [0.9195-0.9596]	0.9554 [0.94-0.97]	0.8168 [0.7174-0.8837]	0.8040 [0.6987-0.8752]	0.8951 [0.83-0.94]	0.9358 [0.9079-0.9554]	0.9251 [0.8928-0.9479]	0.9666 [0.95-0.98]	0.8683 [0.7650-0.9280]	0.8681 [0.7647-0.9279]	0.9157 [0.84-0.96]	0.8871 [0.8249-0.9280]	0.9315 [0.8924-0.9567]	0.9355 [0.90-0.96]
CPV	0.9298 [0.8897-0.9556]	0.9406 [0.9065-0.9626]	0.9517 [0.92-0.97]	0.9325 [0.9052-0.9522]	0.9338 [0.9069-0.9531]	0.9499 [0.93-0.96]	0.8922 [0.8298-0.9325]	0.9089 [0.8554-0.9432]	0.9367 [0.90-0.96]	0.9235 [0.8905-0.9468]	0.9249 [0.8925-0.9478]	0.9590 [0.94-0.97]	0.8273 [0.6970-0.9047]	0.9094 [0.8355-0.9510]	0.9017 [0.82-0.95]	0.8613 [0.7865-0.9111]	0.9184 [0.8724-0.9483]	0.9211 [0.87-0.95]
NCPV	0.8366 [0.7504-0.8949]	0.7842 [0.6752-0.8597]	0.8879 [0.82-0.93]	0.8825 [0.8365-0.9161]	0.9015 [0.8624-0.9299]	0.9339 [0.91-0.95]	0.7097 [0.5660-0.8115]	0.6446 [0.4785-0.7662]	0.8071 [0.69-0.88]	0.8449 [0.7820-0.8908]	0.8432 [0.7796-0.8896]	0.9159 [0.88-0.94]	0.8800 [0.7848-0.9346]	0.8542 [0.7413-0.9201]	0.9197 [0.85-0.96]	0.8771 [0.8101-0.9215]	0.9002 [0.8446-0.9365]	0.9285 [0.89-0.96]
LAPV	0.2379 [0.0066-0.4450]	0.1443 [-0.0904-0.3638]	0.2750 [-0.16-0.55]	0.2894 [0.1199-0.4425]	0.3771 [0.2157-0.5183]	0.3214 [0.03-0.52]	0.0440 [-0.1983-0.2812]	0.1218 [-0.1219-0.3517]	0.0382 [-0.56-0.41]	0.2061 [0.0214-0.3772]	0.2837 [0.1035-0.4457]	0.1683 [-0.21-0.43]	0.2161 [-0.0981-0.4911]	0.3418 [0.0382-0.5877]	0.1704 [-0.56-0.56]	0.2085 [-0.0243-0.4199]	0.3704 [0.1517-0.5545]	0.1939 [-0.29-0.50]
LV	0.9318 [0.8928-0.9569]	0.9191 [0.8734-0.9488]	0.9474 [0.92-0.97]	0.9304 [0.9022-0.9507]	0.9561 [0.9379-0.9690]	0.9491 [0.93-0.96]	0.8780 [0.8082-0.9234]	0.8986 [0.8396-0.9366]	0.8978 [0.83-0.94]	0.8975 [0.8542-0.9284]	0.9018 [0.8601-0.9315]	0.9332 [0.90-0.95]	0.9485 [0.9048-0.9724]	0.9113 [0.8389-0.9520]	0.9647 [0.93-0.98]	0.9371 [0.9011-0.9603]	0.9572 [0.9322-0.9731]	0.9489 [0.92-0.97]
VV	0.9233 [0.8798-0.9515]	0.9256 [0.8833-0.9529]	0.9440 [0.91-0.96]	0.9464 [0.9244-0.9621]	0.9679 [0.9546-0.9774]	0.9641 [0.95-0.97]	0.8957 [0.8351-0.9348]	0.9027 [0.8459-0.9393]	0.9175 [0.87-0.95]	0.9538 [0.9335-0.9680]	0.9437 [0.9191-0.9610]	0.9737 [0.96-0.98]	0.9313 [0.8740-0.9630]	0.9380 [0.8860-0.9667]	0.9533 [0.91-0.98]	0.9223 [0.8783-0.9508]	0.9571 [0.9322-0.9730]	0.9492 [0.92-0.97]

Plaque Correlations by kVP

Patients	kVP 120 (n=145)						kVP 100 (n=58)					
	Per-Vessel (n=151)			Per-Lesion (n=261)			Per-Vessel (n=59)			Per-Lesion (n=113)		
Variables	Vessel Pearson	Vessel Spearman	ICC Vessel	Lesion Pearson	Lesion Spearman	ICC Lesion	Vessel Pearson	Vessel Spearman	ICC Vessel	Lesion Pearson	Lesion Spearman	ICC Lesion
TPV	0.7918 [0.7236-0.8447]	0.7890 [0.7201-0.8426]	0.8769 [0.83-0.91]	0.9076 [0.8836-0.9269]	0.9193 [0.8982-0.9362]	0.9494 [0.94-0.96]	0.8743 [0.7966-0.9236]	0.8667 [0.7849-0.9189]	0.9211 [0.87-0.95]	0.9237 [0.8910-0.9468]	0.9318 [0.9024-0.9525]	0.9588 [0.94-0.97]
CPV	0.8862 [0.8463-0.9162]	0.9216 [0.8933-0.9425]	0.9325 [0.91-0.95]	0.9077 [0.8837-0.9270]	0.9259 [0.9064-0.9415]	0.9465 [0.93-0.96]	0.9411 [0.9025-0.9647]	0.9325 [0.8886-0.9595]	0.9541 [0.92-0.97]	0.9304 [0.9004-0.9516]	0.8410 [0.7770-0.8878]	0.9523 [0.93-0.97]
NCPV	0.7448 [0.6641-0.8084]	0.7260 [0.6406-0.7937]	0.8401 [0.78-0.88]	0.8618 [0.8269-0.8900]	0.8732 [0.8409-0.8992]	0.9222 [0.90-0.94]	0.8584 [0.7721-0.9137]	0.7725 [0.6438-0.8587]	0.8999 [0.83-0.94]	0.8869 [0.8397-0.9207]	0.8924 [0.8473-0.9247]	0.9352 [0.91-0.96]
LAPV	0.1353 [-0.0250-0.2887]	0.2008 [0.0424-0.3493]	0.1431 [-0.18-0.38]	0.2560 [0.1389-0.3660]	0.3811 [0.2723-0.4803]	0.2723 [0.07-0.43]	0.3368 [0.0884-0.5458]	0.3428 [0.0951-0.5505]	0.3569 [-0.08-0.62]	0.3628 [0.1908-0.5131]	0.4977 [0.3446-0.6249]	0.3363 [0.04-0.54]
LV	0.9209 [0.8924-0.9421]	0.9063 [0.8730-0.9312]	0.9439 [0.92-0.96]	0.9382 [0.9218-0.9513]	0.9527 [0.9400-0.9627]	0.9519 [0.94-0.96]	0.9047 [0.8442-0.9425]	0.9392 [0.8995-0.9635]	0.9274 [0.88-0.96]	0.9351 [0.9071-0.9549]	0.9375 [0.9104-0.9565]	0.9420 [0.92-0.96]
VV	0.9039 [0.8698-0.9294]	0.8995 [0.8639-0.9262]	0.9363 [0.91-0.95]	0.9442 [0.9293-0.9560]	0.9551 [0.9430-0.9646]	0.9625 [0.95-0.97]	0.9358 [0.8940-0.9615]	0.9293 [0.8835-0.9575]	0.9483 [0.91-0.97]	0.9585 [0.9402-0.9713]	0.9513 [0.9299-0.9662]	0.9684 [0.95-0.98]

Bland-Altman Plots

Bland-Altman Plot (Siemens Somatom Force)

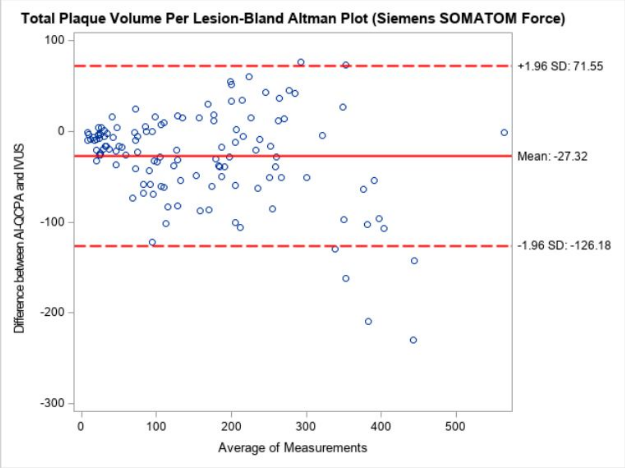

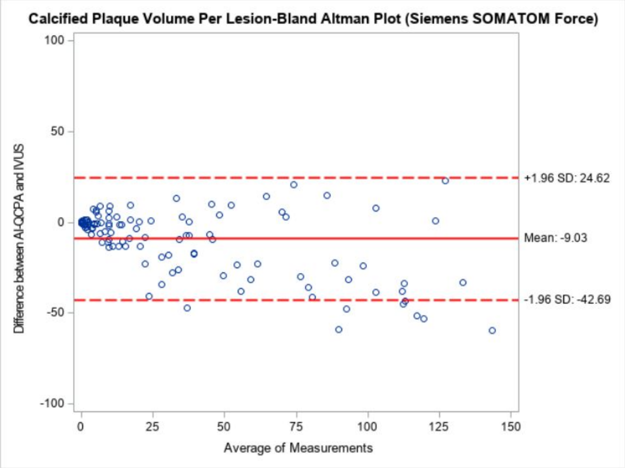

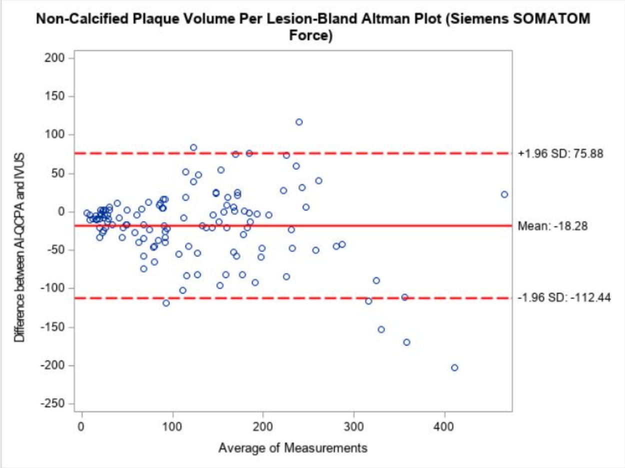

Bland-Altman Plot (GE Revolution)

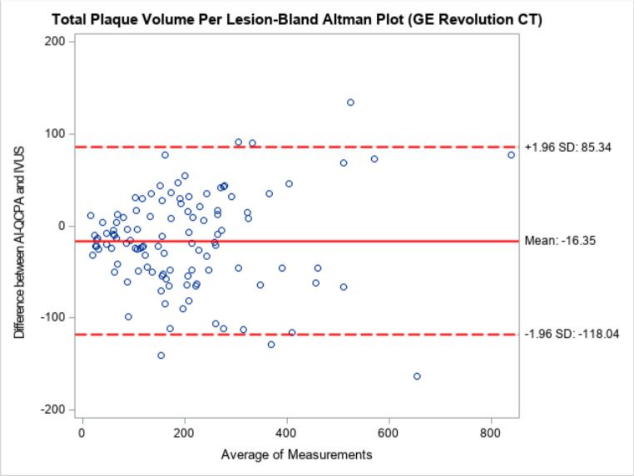

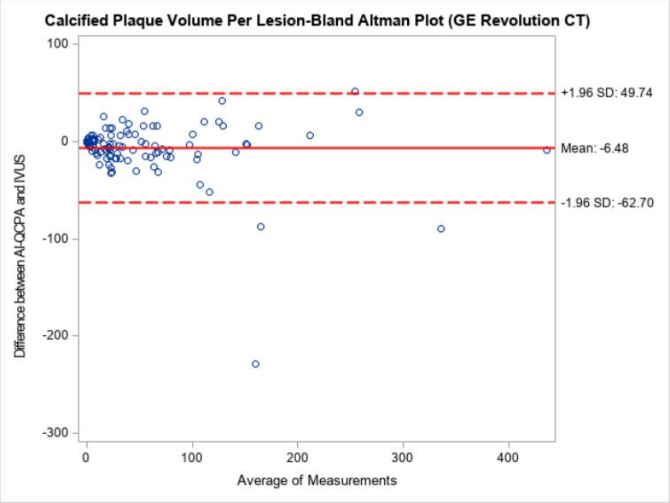

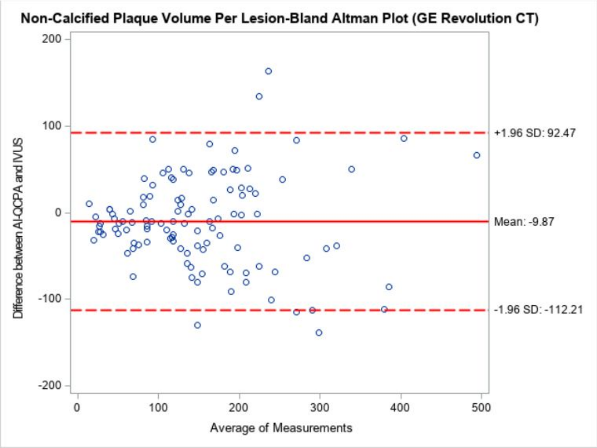

Bland-Altman Plot (Canon Aquilion ONE)

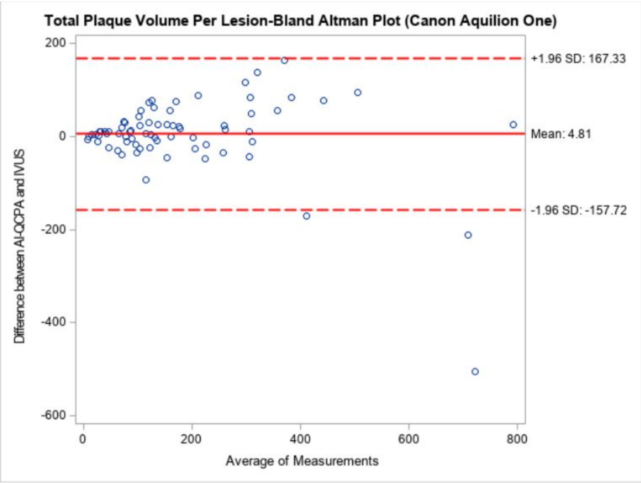

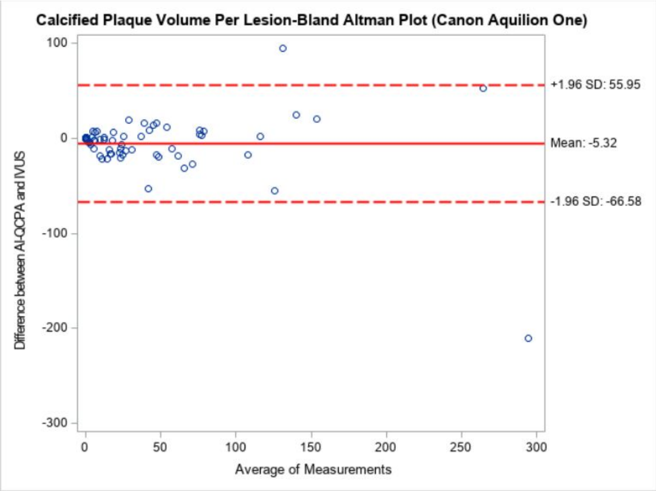

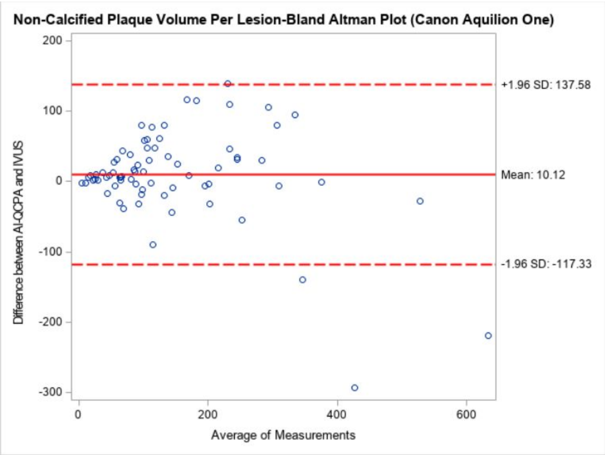

Bland-Altman Plot (120 kVP)

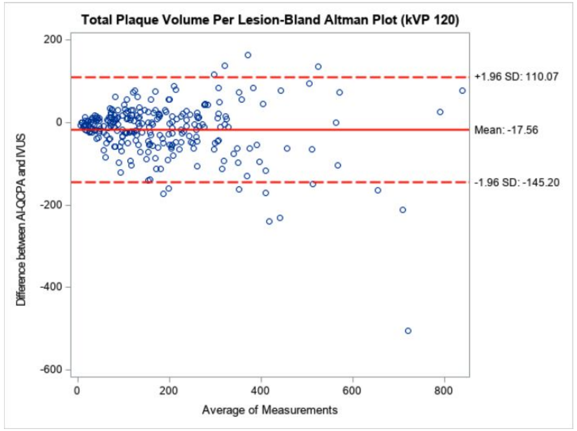

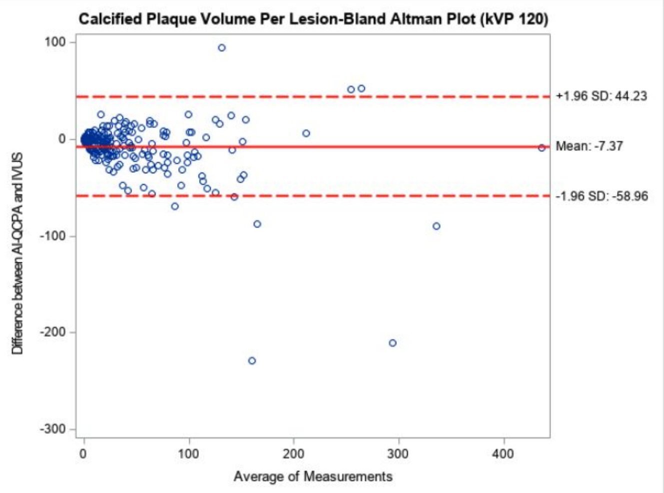

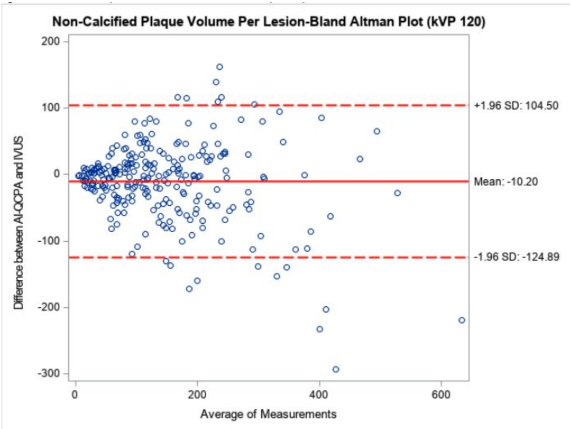

Bland-Altman Plot (100 kVP)

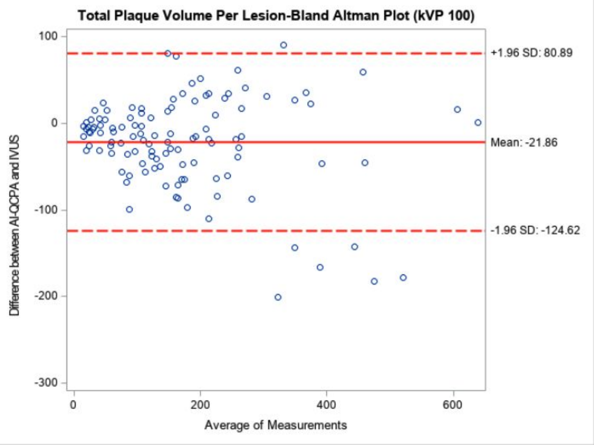

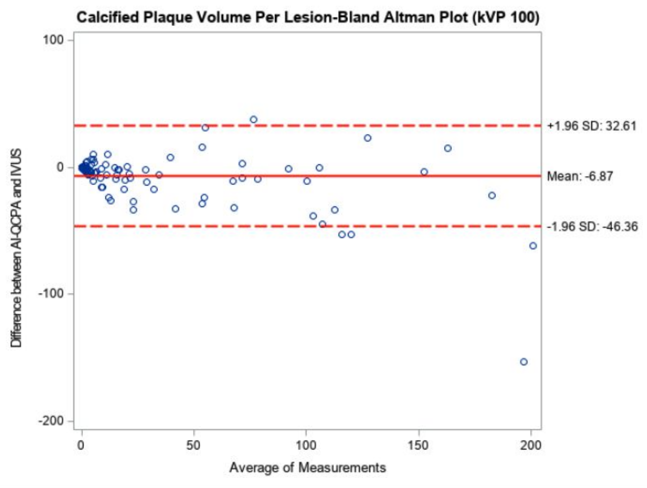

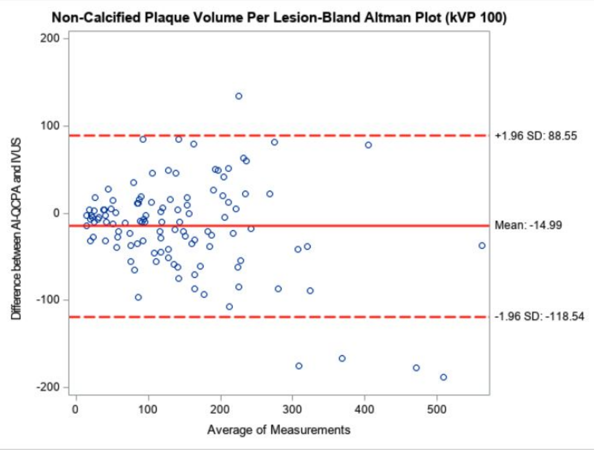

Supplement: jeae115_Supplementary_Data [file jeae115_supplementary_data.zip › plots_April 5.pdf]
